# Long-Term Effects of Dietary Olive Oil and Hydrogenated Vegetable Oil on Expression of Lipogenic Genes in Subcutaneous Adipose Tissue of Dairy Cows

**DOI:** 10.3390/vetsci6030074

**Published:** 2019-09-15

**Authors:** Einar Vargas-Bello-Pérez, Massimo Bionaz, Pietro Sciarresi-Arechabala, Nathaly Cancino-Padilla, María Sol Morales, Jaime Romero, Heidi Leskinen, Philip C. Garnsworthy, Juan J. Loor

**Affiliations:** 1 Departamento de Ciencias Animales, Facultad de Agronomía e Ingeniería Forestal, Pontificia Universidad Católica de Chile, Santiago Casilla-306, Chile; nlcancino@uc.cl; 2 Department of Veterinary and Animal Sciences, Faculty of Health and Medical Sciences, University of Copenhagen, Grønnegardsvej 3, C DK-1870 Frederiksberg, Denmark; 3 Department of Animal and Rangeland Sciences, Oregon State University, Corvallis, OR 97331, USA; 4 Departamento de Fomento de la Producción Animal, Facultad de Ciencias Veterinarias y Pecuarias, Universidad de Chile. Av. Santa Rosa 11735, La Pintana, Santiago 8820808, Chile; pietro.sciarresi@ug.uchile.cl (P.S.-A.); smorales@uchile.cl (M.S.M.); 5 Laboratorio de Biotecnología en Alimentos, Unidad de Alimentos, Instituto de Nutrición y Tecnología de los Alimentos, Universidad de Chile, Avda. El Libano 5524, Macul, Santiago, 7830490, Chile; jromero@inta.uchile.cl; 6 Milk Production, Production Systems, Natural Resources Institute Finland (Luke), 31600 Jokioinen FI, Finland; heidi.leskinen@luke.fi; 7 School of Biosciences, The University of Nottingham, Sutton Bonington Campus, Loughborough LE12 5RD, UK; Phil.Garnsworthy@nottingham.ac.uk; 8 Department of Animal Sciences and Division of Nutritional Sciences, Mammalian NutriPhysioGenomics, University of Illinois, Urbana, IL 61801, USA; jloor@illinois.edu

**Keywords:** fat supplementation, transcriptomics, adipose tissue, lipid metabolism, lipogenic gene expression, dairy cows

## Abstract

The objective of this study was to characterize the long-term transcriptomic effects of lipogenic genes in subcutaneous adipose tissue (SAT) of dairy cows supplemented with unsaturated (olive oil; OO) and saturated (hydrogenated vegetable oil; HVO) lipids. Cows were fed a control diet with no added lipid, or diets containing OO or HVO (n = 5 cows/group) for 63 days. SAT was obtained from the tail-head area at the onset of the study and after 21, 42, and 63 days of supplementation. Treatments had minor effects on expression of measured genes. Both fat supplements reduced expression of *PPARG*, HVO decreased transcription of the desaturase *FADS2* and lipid droplet formation *PLIN2*, and OO increased transcription of *FABP3*. Both lipid treatments decreased expression of the transcription regulator *SREBF1* and its chaperone (*SCAP*) during the first 21 days of treatment. Our data indicated that long-term feeding of OO and HVO have a relatively mild effect on expression of lipogenic genes in SAT of mid-lactating cows.

## 1. Introduction

Long-chain fatty acids (LCFA) are the only dietary compounds with evident nutrigenomic effects in dairy cows, especially through the Peroxisome Proliferator-activated Receptors [1,2]. Some of the first evidence of in vivo nutrigenomic roles of LCFA was observed in conjugated linoleic acids. As intermediate products of rumen biohydrogenation of polyunsaturated LCFA, they negatively affect expression of lipogenic genes in mammary tissue of dairy cows [3]. Further in vivo evidence was accumulated by feeding LCFA to dairy cows with transcriptomic effects observed in mammary [4] and adipose tissue [5]. In vitro data clearly indicated that saturated LCFA have stronger nutrigenomic effects compared to unsaturated LCFA in ruminants, especially C16:0 and C18:0 [2,6]. Among unsaturated LCFA, C18:1 has the least nutrigenomic effect as observed in vitro in bovine mammary cells [7] and in vivo in calf’s liver [8]. However, C18:1 has important functions in animals, as exemplified by the key role of stearoyl-CoA desaturase (*SCD1*) [9], including in milk fat synthesis [10]. Some evidence of an effect of dietary supplementation of C18:1 in dairy cows was provided by our recent study [11]. Compared to a non-supplemented diet, supplementing olive oil to a total mixed ration reduced milk fat yield while increasing proportions of several unsaturated LCFA in milk.

Lipogenesis in mammary gland is a consequence of de novo fatty acid synthesis and absorption of pre-formed fatty acids. Besides mammary gland, the most important lipogenic tissue is the adipose. Thus, subcutaneous adipose tissue (SAT) can compete with the mammary gland for LCFA and other precursors affecting milk fat synthesis. This was evidenced by increased expression of lipogenic genes in dairy cows with milk fat depression [12]. Thus, the reduction of milk fat observed in our prior study [11] may be a consequence of increased lipogenesis in adipose tissue. Few in vivo studies have evaluated the nutrigenomic effects of supplementing fat in adipose tissue of dairy cows. Also, earlier studies which characterized gene expression in SAT of cows subjected to lipid supplementation, normally lasted only up to four weeks [5,13]. Fat in the diet of dairy cows is normally supplemented for more than four weeks; thus, the long-term response to LCFA supplementation remains to be determined.

The objective of this study was to characterize the long-term transcriptomic effects of lipogenic genes in SAT of dairy cows supplemented with unsaturated (olive oil; OO) and saturated (hydrogenated vegetable oil; HVO) lipids for 9 weeks. Our hypothesis is that both lipid supplements affect expression of lipogenic genes in SAT with a higher effect of HVO compared to OO.

## 2. Materials and Methods

### 2.1. Animals, Experimental Diets and Tissue Sampling

Animal care and procedures were carried out according to the guidelines of the Animal Care Committee of the Pontificia Universidad Católica de Chile. The study was conducted at the Estación Experimental Pirque of the Pontificia Universidad Católica de Chile (project code 150730013).

Details of the study were previously published [11]. Briefly, fifteen cows (189 ± 28 days in milk and 36.8 ± 3.2 kg/d of milk) received a basal diet (65% forage and 35% concentrate) distributed as a total mixed ration. The control or basal diet contained no added lipid (n = 5 cows); treatment diets were supplemented with OO (n = 5 cows; unrefined olive oil; 30 g/kg DM) or HVO (n = 5 cows; manufactured from palm oil; 30 g/kg DM) for 63 days. Oils were mixed manually into the daily ration for each cow.

### 2.2. Biopsies, RNA Extraction, and Reverse Transcription Quantitative Polymerase Chain Reaction (RTqPCR)

Samples of SAT tissue were collected from the tail-head of each cow at the start of the experiment (day 0) and on days 21, 42, and 63 of supplementation, using the biopsy method described in [5]. Biopsied tissue (1–2 g) was cut into small slices (<0.5 cm thick), immediately submerged in 5 volumes of RNA stabilization reagent (RNAlater; Sigma, Cat. No. R0901, St Louis, MI, USA), maintained at 4 °C for 24 h and then stored at −80 °C before until RNA extraction.

RNA extraction was performed with QIAzol Lysis Reagent (Qiagen Inc., Valencia, CA, USA) following the manufacturer’s instructions. Quality and quantity of RNA were evaluated by gel electrophoresis with 1% agarose and RNA quantification was measured fluorometrically using the Qubit RNA HS Assay Kit in the Qubit Fluorometer 3.0 (Invitrogen Co., Carlsbad, CA, USA) (Figure S1). To avoid genomic DNA amplification, samples were treated with RQ1 RNase-Free DNase (Cat. No. M6101; Promega^®^, Madison, WI, USA), and confirmation of genomic DNA removal was established by polymerase chain reaction (PCR).

Synthesis of first-strand cDNA was performed on a SureCycler 8800 Thermal Cycler (Agilent Technologies Inc., Santa Clara, CA, USA) and running the ImProm-II^®^ Reverse Transcription System (Promega^®^, Madison, WI, USA). Total RNA was mixed with 0.5 µg per reaction oligo (dT)15 primer (Cat. No. C1101; Promega^®^, Madison, WI, USA) to give a final volume of 5 µL, which was incubated for 5 min at 70 °C. Then 15 µL of transcription mix (0.5 mM of Recombinant RNasin^®^ Ribonuclease Inhibitor, 0.5 mM of dNTP and 2.25 mM of MgCl_2_ in 4.6 µL of ImProm-II 5 × Reaction Buffer (Promega, Cat. No. N2511, Madison, WI, USA) with a volume of 0.5 µL, and 1 µL ImProm-II Reverse Transcriptase (Promega, Cat. No. A3802, Madison, WI, USA) was added. After adding the transcription mix, the reaction was incubated for 5 min at 25 °C and then incubated for 60 min at 42 °C. To stop the reverse transcription reactions, the mixture was heated for 15 min at 70 °C. Due to quality and quantity of extracted RNA, a subgroup of nine cows (three per group) were used for gene expression analysis.

Genes tested in the current study and their lipogenic role are listed in Table 1. Primer-pairs were as previously reported [10]. Quantitative PCR analysis was performed using AriaMx^®^ (Agilent Technologies, Santa Clara, CA, USA). The amplification of specific PCR products was performed using LightCycler 480 SYBR Green I Master ^®^ (Roche, Cat. No 4887352001, Indianapolis, IN, USA) according to the manufacturer’s instructions. All cDNA samples were analyzed in triplicate. The amplification protocol was as follows: one initial step at 95 °C for 10 min (denaturation and enzyme activation) followed by 40 cycles at 95 °C for 15 s, 60 °C for 1 min. After amplification, a melting curve analysis was performed over a range of 65 °C–95 °C to verify that a single PCR product was generated at the end of the assay. The final PCR data were calculated using LinRegPCR 12.18 software [14].

### 2.3. Statistical Analysis

*GAPDH* (glyceraldehyde 3-phosphate dehydrogenase), *EIF3K* (eukaryotic translation initiation factor 3 subunit K), and *UXT* (ubiquitously expressed prefoldin like chaperone) were tested as reference genes by the geNorm algorithm [15]. All three genes had an M-value of ≤0.80 and use of the geometrical mean of the three reference genes provided a V-value of 0.245, indicating a good normalization factor.

Prior to statistical analysis, data were transformed as fold change relative to the mean of the HVO group at time 0 for each gene, as previously described [16]. Outliers were checked using Proc Reg of SAS (v.9.4, SAS Institute Inc., Cary, NC, USA) removing data with a studentized t > 3.0 prior to statistical analysis. Final data were analyzed using Proc GLIMMIX of SAS with Diet, Time, and Diet × Time interaction as main effects and cow as a random effect. The Spatial Power was used as covariate model as per the inequal time distribution. Due to the low number of animals per group (n = 3) a *p* ≤ 0.10 was declared as significant and *p* ≤ 0.15 as tendency. A *p* ≤ 0.05 was used as post-hoc differences. Correlation analysis was performed using Proc Corr of SAS.

## 3. Results

### 3.1. Lipogenic Genes Affected by OO or HVO

A summary view of transcription of lipogenic genes measured in SAT of cows treated with olive oil or hydrogenated olive oil is shown in Table 2. Few genes were affected by treatments in each functional category. Among genes related to fatty acid transport only *FABP3* was overall more expressed in HVO versus CON. *FADS2* was the only transcript related to de novo synthesis of LCFA with higher expression in OO versus HVO. Of transcripts related to triacylglycerol synthesis and lipid droplet formation, only *PLIN2* was more expressed in HVO versus CON. *PPARG* was the only mRNA among the ones related to transcription regulation to be overall affected by diet, with lower expression in OO compared to CON.

#### 3.1.1. LCFA Transport and Activation

**Figure 1:**
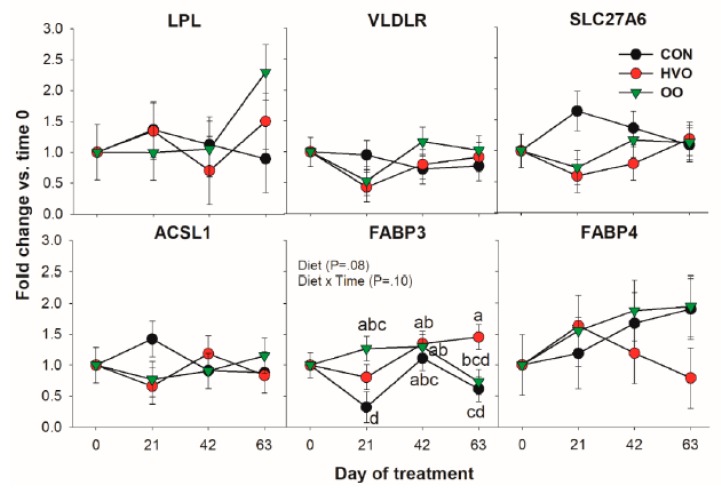
Effect of olive oil (OO) or hydrogenated olive oil (HVO) on transcripts coding for proteins involved in fatty acid transport and activation. Reported in the graph are the *p*-value of the overall effect of Diet and Diet × Time interaction. Diverse letters denote significant post-hoc differences (*p* < 0.05).

In Figure 1, the pattern for all the transcripts coding for proteins involved in LCFA transport and activation is reported. Among genes measured in this category, only *FABP3* was significantly affected by the treatments. Compared to CON, OO had higher expression of *FABP3* at 21 day, and HVO was higher than OO at 63 days of treatment.

#### 3.1.2. De Novo Fatty Acid Synthesis

**Figure 2:**
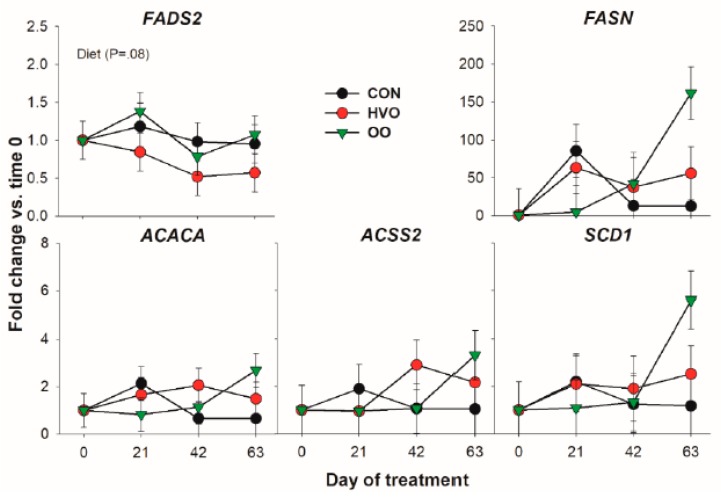
Effect of olive oil (OO) or hydrogenated olive oil (HVO) on transcripts coding for proteins involved in de novo fatty acid synthesis. Reported in the graph are the *p*-value of the overall effect of Diet.

Except for an overall higher transcription of *FADS2* in OO versus HVO, none of the genes coding for proteins involved in de novo fatty acid synthesis was affected by the treatments (Figure 2). Although none reached significance, there was a similar numerical increase in expression for all the genes in this category after 63 days of OO treatment.

#### 3.1.3. Triacylglycerol Synthesis and Lipid Droplet Formation

**Figure 3:**
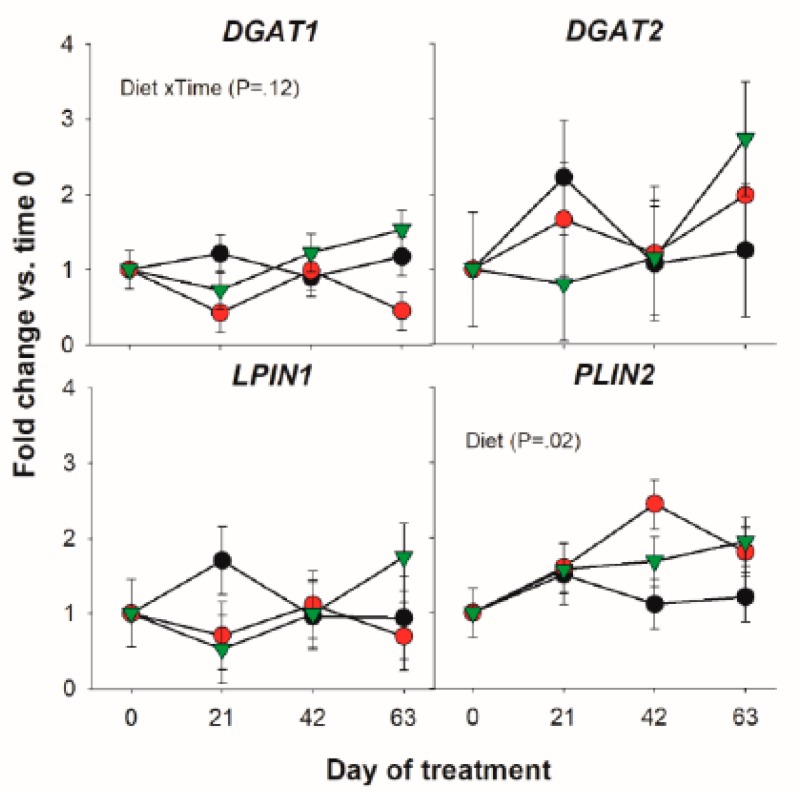
Effect of olive oil (OO) or hydrogenated olive oil (HVO) on transcripts coding for proteins involved in triacylglycerol synthesis. Reported in the graph are the *p*-value of the overall significant effect of Diet and tendency for the effect of Diet × Time interaction.

Among transcripts coding for proteins involved in triacylglycerol synthesis only a tendency for a Diet × Time interaction for the *DGAT1* was detected (Figure 3).

#### 3.1.4. Transcription Regulation

**Figure 4:**
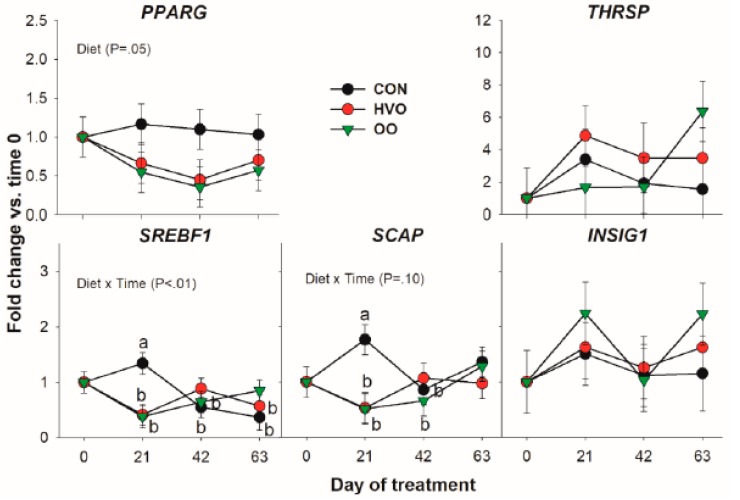
Effect of olive oil (OO) or hydrogenated olive oil (HVO) on transcripts coding for proteins involved in transcription regulation of lipid-related genes. Reported in the graph are the *p*-value of the overall effect of Diet and Diet × Time. Diverse letters denote significant post-hoc differences (*p* < 0.05).

Transcription of the key de novo acid transcription regulator *SREBF1* and its chaperone *SCAP* were both affected by Diet × Time due to OO and HVO treatment preventing the increase in expression 21 days into the trial. Apart from this, and a lower expression of *PPARG* by OO and HVO, no difference was observed for this category of genes (Figure 4).

#### 3.1.5. Correlation Between Transcripts

**Figure 5:**
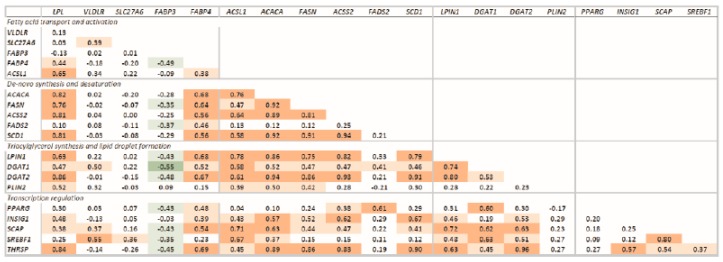
Correlations of the transcription of measured genes. Significant correlations are denoted by bold font and red (positive correlation; *p* < 0.0001), light red (positive correlation: *p* < 0.05), green (negative correlation; *p* < 0.0001), and light green (negative correlation: *p* < 0.05). Transcripts were grouped based on function.

All the measured transcripts were highly correlated, except for *SLC27A6*, *VLDLR*, *PPARG*, and *PLIN2*, which had low correlations with the other transcripts (Figure 5). Transcription of *FABP3* was negatively correlated with transcription of most of the genes measured.

## 4. Discussion

### 4.1. Cow’s Performance

Fat is supplemented in the diets of dairy cows with the main purpose of increasing energy density to sustain milk production. The use of saturated lipid sources is often preferred over the use of oils because oils tend to induce milk fat depression and prevent cellulose degradation in the rumen [17]. However, among unsaturated LCFA, C18:1 is less likely to induce milk fat depression and has the least effect on the transcriptome of mammary tissue. The approach of this study was to feed a modest amount of supplemental oil to cows in order to observe long-term changes in the milk FA profile without affecting animal performance and milk fat concentration. Performance of the animals and milk composition were previously reported [11]. Briefly, body condition score and body weight were not affected by treatments. Compared with control and HVO, OO significantly increased milk yield and reduced milk fat yield. Therefore, the reason for the milk fat depression as consequence of olive oil supplementation in our study remains to be determined. It is possible that the decrease in milk fat observed for OO was partly due to an effect of the treatment on expression of lipogenic genes in the adipose tissue.

### 4.2. Lipid Supplements Might Have Anti-Lipolitic and Anti-Adipogenic Effect

Contrary to our initial hypothesis, few genes were affected by HVO and OO. None of the data indicated any increase in lipogenesis in adipose tissue as consequence of lipid supplement. Data might indicate decrease of adipogenesis with lipid supplementation by decreasing expression of PPARγ. This transcript is a key regulator of adipogenesis and lipogenesis in SAT of ruminants and non-ruminants [18]. Along with this, the activity of PPARγ can be modulated by LCFA. Targets of PPARγ include several lipogenesis-related genes [1,19]. Although a significant decrease in expression of PPAR γ was detected, we did not observe any significant effect on putative target genes in our study, indicating that the reduction of the amount of transcription factor did not reduce its activation. Support for this is also found in the relatively low correlation of PPARγ transcription with lipogenic-related genes measured. It is possible that the large amount of the putative PPARγ activators C16:0 and C18:0 [1] in lipid treatments maintained the activation of PPARγ compensating for its reduced abundance compared to the unsupplemented cows.

Another transcription factor affected in our study was the key de novo FA synthesis regulator SREBF1 [2]. Interestingly, expression of its chaperone SCAP, essential for the transport of SREBP1 to the Golgi for subsequent activation, had a similar pattern. The pattern of transcription of those two genes was peculiar with no alteration in the temporal pattern of transcription by the two lipid supplements but an unexplained increase at 21 days into the experiment in the CON group. If the pattern of SREBF1 and SCAP transcription in the CON was the expected one, the two lipid supplements prevented the increase in transcription of the de novo FA regulator. The observed changes did not have any significant effect on transcription of the SREBP1 target genes FASN and SCD1, although having a similar pattern also supported by the high correlation between SREBF1 and these genes. The lack of significant effect on de novo synthesis genes was likely due to insufficient statistical power.

PLIN2 codes for a protein involved in lipid storage and lipid droplet formation, its overexpression being related to accumulation of lipids in SAT [20]. Although a general increase in its expression was observed during the period of lipid supplementation, the observed effect was greater in the HVO group. Greater expression of this gene might indicate larger accumulation of lipids in the adipose tissue of lipid-supplemented dairy cows.

The above results indicate possible anti-adipogenic and anti-lipogenic effects of lipid supplements. The latter appeared more marked in the case of HVO as indicated by the pattern of DGAT1 and FADS2. Due to lack of significant effect on other genes related to lipid synthesis and a possible anti-lipogenic effect inferred by the transcription factors, it is unclear the consequence of the observed increase in expression of PLIN2. The lack of effects on body condition score observed [11] support the absence of effects on lipogenesis.

### 4.3. Long-Term Supplementation of Lipids Does Not Improve the Nutrigenomic Effect of Lcfa in Mid-Lactation Cows

Our results are somewhat similar to previous studies with similar conditions, for example, no difference was observed in expression of DGAT1 or PPAR γ in adipose tissue of mid-lactating cows supplemented with 3% DM (same amount of lipid supplement used in this study) of dietary saturated fat for 21 days [5]. However, in the same study mRNA expression of several lipogenic genes was upregulated in SAT of cows fed soybean oil for 21 days. Expression of genes related to lipogenesis in mammary tissue of cows and goats supplemented with palm oil for 28 days has been evaluated [21]. Although a positive effect on milk fat concentration was observed, there was no change in expression of any measured gene. On the other hand, a previous study reported an increase in expression of genes involved in de novo FA synthesis, desaturation, triglyceride synthesis and the transcription factor SREBF1 after seven days of feeding mid-lactation cows with 3% DM of a commercial lipid supplement enriched with C16:0 compared to fish/soybean oil [4]. Effects disappeared, however, after 21 days of supplementation.

Stage of lactation might also play a role. A pro-lipogenic effect was reported in adipose tissue of cows during the peripartum period by supplementing with saturated lipid sources [13]. This was inferred based on expression of PPAR γ and several of its coregulators and putative target genes. The difference between the latter study and ours can be partly explained by stage of lactation but also by the FA composition of the supplements administered. In the prior study [13] a lipid supplement containing 29% C16:0 and 55% C18:0 was used. In our trial, the supplement had 58% C16:0 and 40% C18:0. This is especially interesting because of the possible differences in the diet of C16:0, since apparently this would have a greater nutrigenomic effect within the saturated long-chain FA [2].

Evidence of larger nutrigenomic effects of C16:0 versus C18:0 come from in vitro studies in SAT cells and mammary gland epithelial cells [7,22,23]. Within these studies, the effect of C18:1 on transcription of PPAR γ and its target genes was also evaluated. Although important lipogenic effects and stimulation in PPAR γ expression after culturing adipose tissue cells together with C18:1 was observed in one study [22], no effect was reported in another study in bovine adipose tissue ex vivo [23]. Our data confirm the findings of the latter study.

Overall, our data, together with published data, indicate that adipose tissue of mid-lactation dairy cows might be less responsive to a nutrigenomic effect of LCFA.

### 4.4. Limitations of The Study

Our results show only marginal changes in mRNA abundance of the measured genes and related transcription factors in adipose tissue. This can be partly due to limitations of our study. The major limitation is the low number of animals used for each treatment as consequence of RNA quality. Another limitation is the lack of other measurements to support findings from transcriptomic data. For instance, size distribution of adipocytes would have helped in determining change in adipogenesis/lipogenesis by the lipid supplements. We also used low amounts of supplemental oils. It is well known that transcription factors, including PPAR in dairy cows, respond in a dose-dependent fashion [24]. This agrees in part with a study where minor effects were observed in adipose tissue of mid-lactation ewes in positive energy balance fed with sunflower oil (25 g/kg DM for 7 weeks) [25].

## 5. Conclusions

Our study only marginally supports the hypothesis that lipid supplements used would have affected expression of lipogenic genes in SAT with a higher effect of HVO compared to OO. However, the study confirmed a mild nutrigenomic effect of olive oil in dairy cows. Surprisingly, only a minor effect was observed for HVO, which is rich in C16:0, a known strong nutrigenomic LCFA. Our data also suggest that SAT tissue of mid-lactation dairy cows might have a low nutrigenomic response.

## Figures and Tables

**Table 1:** Gene symbol, name, and lipogenesis-related functions of the 20 genes measured in the present study.

Symbol	Name	Function
*ACACA*	Acetyl-CoA carboxylase alfa	Catalyzes the rate-limiting reaction in the de novo synthesis of long-chain fatty acids (LCFA)
*ACSL1*	Acyl-CoA Synthetase Long Chain Family Member 1	Convert LCFA into acyl-CoA esters, transport of exogenous fatty acid (FA)
*ACSS2*	Acyl-CoA Synthetase Short Chain Family Member 2	The chemical reactions and pathways resulting in the formation of acetyl-CoA from acetate
*PLIN2*	Adipose Differentiation-Related Protein	Involved in formation and maintenance of lipid droplets
*DGAT1* and *2*	Diacylglycerol O-acyltransferase Homolog 1 and 2	Acyltransferase that catalyzes the terminal and only committed step in triacylglycerol synthesis
*FABP3* and *4*	Fatty Acid Binding Protein 3 and 4	Intracellular transport of acyl-CoA; regulation of gene expression by providing LCFA to PPARγ
*FADS2*	Fatty acid desaturase 2	Desaturase introducing a cis double bond at carbon 6 of the fatty acyl chain
*FASN*	Fatty acid synthase	Fatty acid synthetase catalyzes the formation of long-chain fatty acids from acetyl-CoA, malonyl-CoA and NADPH
*SLC27A6*	Soluble Carrier Protein 27A	LCFA translocation (high uptake); Convert LCFA into acyl-CoA esters
*INSIG1*	Insulin Induced Gene 1	Mediates feedback control of cholesterol synthesis by controlling SCAP and HMGCR
*LPIN1*	Lipin 1	Dephosphorylation of phosphatidate yielding diacylglycerol; Gene expression (PPARα co- factor)
*LPL*	Lipoprotein Lipase	Catalyzes the hydrolysis of triglycerides from circulating chylomicrons and very low-density lipoproteins
*PPARG*	Peroxisome Proliferator Activated Receptor Gamma	Regulate transcription of lipogenic and adipogenic genes.
*SCAP*	SREBP Chaperone	Protein required for cholesterol as well as lipid homeostasis. Chaperone for activation of SREBP1
*SCD1*	Stearoyl-CoA desaturase 1	Desaturase introducing introduce the first double bond into saturated fatty acyl-CoA substrates
*SREBF1*	Sterol Regulatory Element Binding Transcription Factor	Transcriptional regulation of cholesterol synthesis and lipogenesis genes
*THRSP*	Thyroid Hormone Responsive	Nuclear protein which is important in the regulation of lipid metabolism
*VLDLR*	Very Low-Density Lipoprotein Receptor	Binds very low-density lipoproteins assisting LPL

**Table 2:** Effect on the transcription of genes related to lipid metabolism in subcutaneous adipose tissue (SAT) of cows fed with no fat supplement (CON), 30 g/kg DM olive oil (OO), or 30 g/kg DM hydrogenated vegetable oil (HVO).

Gene	CON	HVO	OO	SEM	Diet (D)	Time (T)	D × T
Fatty acid transport and activation							
*LPL*	1.10	1.14	1.34	0.20	0.64	0.47	0.62
*VLDLR*	0.86	0.78	0.93	0.12	0.72	0.27	0.60
*SLC27A6*	1.28	0.89	1.01	0.17	0.34	0.89	0.27
*FABP3*	0.76 ^b^	1.15 ^a^	1.07 ^ab^	0.11	0.08	0.06	0.10
*FABP4*	1.44	1.15	1.59	0.33	0.65	0.43	0.77
*ACSL1*	1.05	0.92	0.93	0.21	0.90	0.97	0.13
De-novo synthesis and desaturation							
*ACACA*	1.11	1.55	1.41	0.52	0.83	0.50	0.19
*FASN*	28.0	39.5	52.4	13.0	0.41	0.15	0.22
*ACSS2*	1.25	1.75	1.58	0.77	0.90	0.59	0.28
*FADS2*	1.03 ^ab^	0.73 ^b^	1.06 ^a^	0.11	0.08	0.34	0.92
*SCD1*	1.40	1.88	2.25	0.83	0.77	0.16	0.34
Triacylglycerol synthesis and lipid droplet formation							
*LPIN1*	1.15	0.88	1.07	0.28	0.78	0.98	0.32
*DGAT1*	1.07	0.72	1.12	0.17	0.26	0.44	0.12
*DGAT2*	1.39	1.46	1.42	0.58	0.99	0.23	0.50
*PLIN2*	1.21 ^b^	1.71 ^a^	1.55 ^ab^	0.13	0.02	0.03	0.43
Transcription regulation							
*PPARG*	1.07 ^a^	0.70 ^b^	0.62 ^b^	0.09	0.05	0.40	0.83
*INSIG1*	1.19	1.38	1.62	0.38	0.73	0.07	0.78
*SCAP*	1.24	0.89	0.86	0.14	0.18	0.46	0.10
*SREBF1*	0.82	0.72	0.72	0.14	0.86	0.04	<0.01
*THRSP*	1.97	3.21	2.68	1.35	0.81	0.13	0.47

^a,b,ab^ Means with different superscripts in the same row are different (*p* < 0.05). SEM, standard error of the mean for diet comparison.

## Supplementary Materials

The following is available online at https://www.mdpi.com/2306-7381/6/3/74/s1, Figure S1. RNA integrity assessment using 1% agarose electrophoresis.
