# Supplementary material for: Long-Term Effects of Dietary Olive Oil and Hydrogenated Vegetable Oil on Expression of Lipogenic Genes in Subcutaneous Adipose Tissue of Dairy Cows
Source: Vet Sci. 2019 Sep 15;6(3):74. doi: 10.3390/vetsci6030074 (PMC6789855; doi:10.3390/vetsci6030074)
Supplement: Supplementary file 1 [file vetsci-06-00074-s001.pdf]

Supplementary Materials

Long-Term Effects of Dietary Olive Oil and Hydrogenated Vegetable Oil on Expression of Lipogenic Genes in Subcutaneous Adipose Tissue of Dairy Cows

Einar Vargas-Bello-Pérez, Massimo Bionaz, Pietro Sciarresi-Arechabala, Nathaly Cancino-Padilla, María Sol Morales, Jaime Romero, Heidi Leskinen, Philip C. Garnsworthy and Juan J. Loor

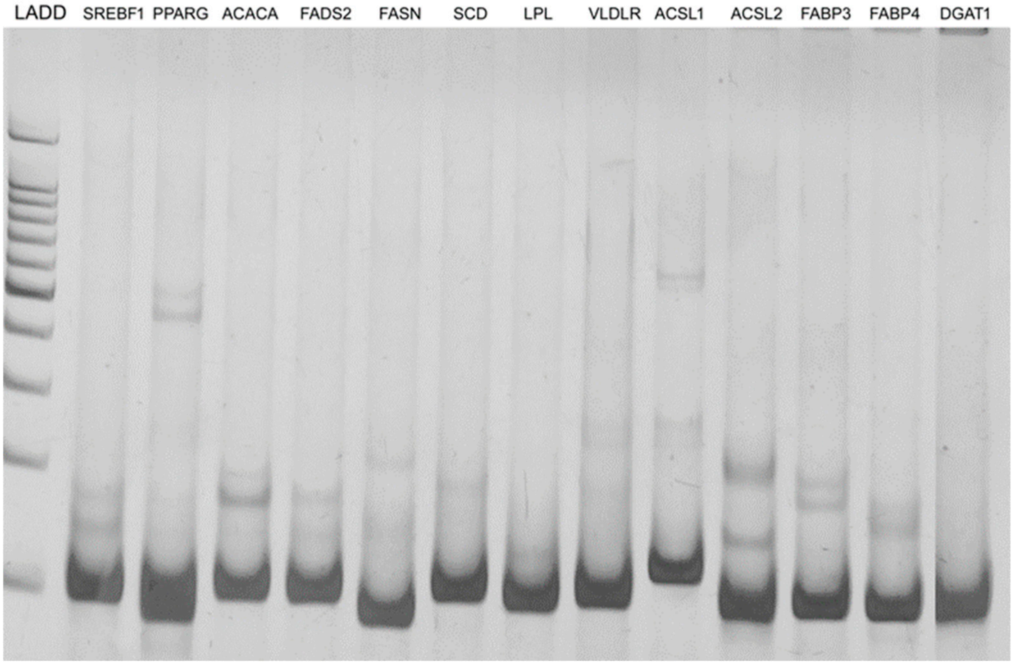

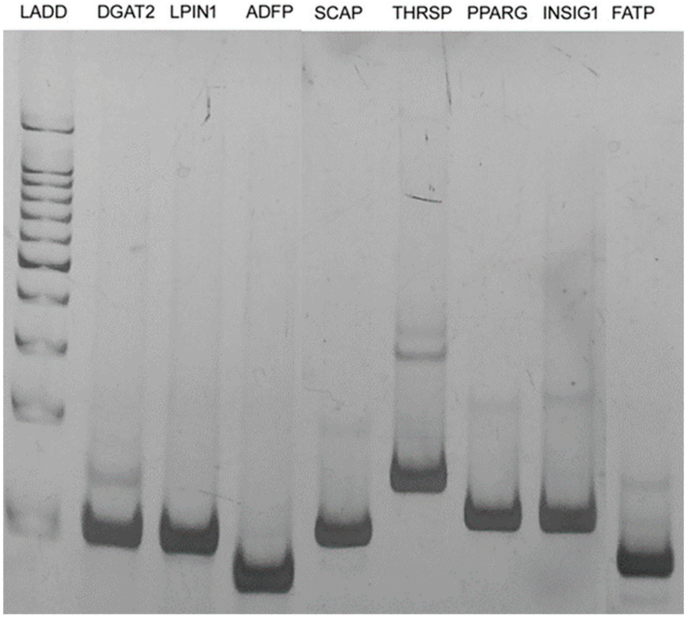

Figure S1. RNA integrity assessment using 1% agarose electrophoresis.

© 2019 by the authors. Licensee MDPI, Basel, Switzerland. This article is an open access article distributed under the terms and conditions of the Creative Commons Attribution (CC BY) license (http://creativecommons.org/licenses/by/4.0/).
